# Nomograms predicting local and distant recurrence and disease-specific mortality for R0/R1 soft tissue sarcomas of the extremities

**DOI:** 10.3389/fonc.2022.941896

**Published:** 2022-09-20

**Authors:** Rita De Sanctis, Renata Zelic, Armando Santoro

**Affiliations:** ^1^ Medical Oncology and Hematology Unit, IRCCS Humanitas Research Hospital, Humanitas Cancer Center, Rozzano, Italy; ^2^ Department of Biomedical Sciences, Humanitas University, Pieve Emanuele, Italy; ^3^ Clinical Epidemiology Division, Department of Medicine, Karolinska Institutet, Stockholm, Sweden

**Keywords:** nomogram, soft tissue sarcoma, perioperative treatment, prognosis, relapse

## Abstract

**Background:**

Prognostic models for patients with soft tissue sarcoma (STS) of the extremities have been developed from large multi-institutional datasets with mixed results. We aimed to develop predictive nomograms for sarcoma-specific survival (SSS) and, for the first time, long-term local recurrence (LR) and distant recurrence (DR) in patients with STS of the extremities treated at our institution.

**Patients and methods:**

Data from patients treated at Humanitas Cancer Center from 1997 to 2015 were analyzed. Variable selection was based on the clinical knowledge and multivariable regression splines algorithm. Perioperative treatments were always included in the model. Prognostic models were developed using Cox proportional hazards model, and model estimates were plotted in nomograms predicting SSS at 5 and 10 years and LR and DR at 2, 5, and 10 years. Model performance was estimated internally *via* bootstrapping, in terms of optimism-corrected discrimination (Harrell C-index) and calibration (calibration plots).

**Results:**

Data on 517 patients were analyzed. At 5 and 10 years, SSS was 68.1% [95% confidence interval (CI), 63.8–72.1] and 55.6% (50.5–60.3), respectively. LR was 79.1% (95% CI, 75.3–82.4), 71.1% (95% CI, 66.7–75.1), and 66.0% (95% CI, 60.7–70.7) at 2, 5, and 10 years, respectively, whereas DR was 65.9% (95% CI, 61.6–69.9), 57.5% (95% CI, 53.0–61.8), and 52.1% (95% CI, 47.1–56.8) at 2, 5, and 10 years, respectively. SSS nomogram included age, gender, margins, tumor size, grading, and histotype. LR and DR nomograms incorporated mostly the same variables, except for age for DR; LR nomogram did not include gender but included anatomic site. The optimism-corrected C-indexes were 0.73 and 0.72 for SSS at 5 and 10 years, respectively; 0.65, 0.64, and 0.64 for LR at 2, 5, and 10 years, respectively; and 0.68 for DR at 2, 5, and 10 years. Predicted probabilities were close to the observed ones for all outcomes.

**Conclusions:**

We developed and validated three nomograms for STS of the extremities predicting the probability of SSS at 5 and 10 years and LR and DR at 2, 5, and 10 years. By accounting for the perioperative treatment, these models allow prediction for future patients who had no perioperative treatment, thus being useful in the clinical decision-making process.

## 1. Introduction

Soft tissue sarcomas (STSs) of the extremities are rare malignant neoplasms accounting for around 1%–2% of all adult cancers. STSs are a heterogeneous group comprising more than 70 different histological subtypes that can manifest at almost any anatomical site (1, 2). Such diversity in STS presentation leads to difficult management and variable prognosis. Indeed, localized STSs of extremities have a 5-year overall survival ranging from 44% to 90% depending on the histological type (3–7).

Localized STSs are typically treated with a combination of surgery and radiotherapy (8, 9). The role of adjuvant or neoadjuvant chemotherapy in STS treatment is controversial due to the conflicting randomized controlled trials’ results (10), but it can be regarded as a relevant option for patients with high-risk STS (9). Treatment decision in STS is currently based on grading, tumor depth, and dimension, which are the main elements of the American Joint Committee on Cancer Staging System for soft tissue sarcoma (9). However, the clinical stage alone is not sufficient to assess patient risk of recurrence or death; furthermore, the therapeutic decision-making process in sarcomas is complicated by the wide molecular heterogeneity within the histological subtypes and by a relative lack of prognostic and predictive biomarkers. Because of the increasing need for individually tailored therapies based on prognostic markers, a more precise risk classification is eagerly needed.

Recently, several nomograms have been developed for predicting long-term outcomes (relapse, overall, and sarcoma-specific survival) in patients with STS (11–23). Many of them focused on specific sites (i.e., primary STS of the trunk and extremity) (14, 18, 19) and/or on specific histological subtypes (14–16, 19, 20). However, with the exception of age, histology, grading, and size, each nomogram identified further different predictors. The variability in the identified prognostic factors may limit the clinical utility of these prediction tools. Furthermore, all these models were developed in additionally treated populations (perioperative chemotherapy and/or radiotherapy), and outcome probabilities, e.g., survival probability, estimated from these models would be overestimated given an effective treatment (24). Only a few of these nomograms included perioperative treatment in the model (13, 15, 20, 23), and, in those who have, treatment was included as yet another predictor. Because none of these studies were designed with estimation of causal effect of perioperative treatment in mind and covariate selection was not following causal inference principles (25, 26), using estimates from these models to compare the probabilities of outcome given one treatment with that of no treatment or another treatment could be highly misleading if used for clinical decision-making.

In the present study, we aimed to develop and internally validate nomograms predicting sarcoma-specific survival (SSS), local recurrence (LR), and distant recurrence (DR) in patients with STS of the extremities surgically treated at our institution—Humanitas Cancer Center, Rozzano, Milan, Italy. By including perioperative treatment in all our models, the estimated outcome probability would reflect the natural history of the disease in untreated individuals (24) and could, in combination with effect estimates for perioperative treatment randomized trials, help clinicians in treatment decision-making.

## 2. Material and methods

### 2.1. Study population, outcome measures, and data collection

All consecutive adult (aged >18 years) patients diagnosed with primary (non-recurrent and non-metastatic) STS of the extremities who were surgically treated at Humanitas Cancer Center (Rozzano, Milan, Italy) from 1997 to 2015 were considered for the inclusion in the study (n = 1,139). Because R2 surgery (i.e., gross residual disease) is considered an inadequate treatment for STS of the extremities, we only included patients with STS receiving R0/R1 surgery. We excluded patients without a histopathological diagnosis of STS of the limbs performed on a biopsy (n = 41) and complete relevant clinical and therapeutic data at baseline (n = 113) and at least 2 years of follow-up (n = 81) to allow for the observation of the relevant outcomes. Furthermore, we excluded desmoid, soft-tissue Ewing’s sarcoma, alveolar or embryonal rhabdomyosarcoma, and dermatofibrosarcoma protuberans because these histological subtypes have a different clinical behavior and are treated differently from other STS of the extremities (n = 261). Finally, we excluded patients with atypical lipomatous tumor/well-differentiated liposarcoma (n = 126) due to their excellent prognosis, because this STS subtype generally does not metastasize neither dedifferentiate at recurrence (7). After the exclusions, 517 patients remained in the study. Data were collected according to an observational protocol that had been approved by the Ethical Committee of Humanitas Research Hospital. Written informed consent to the use of clinical data for scientific purposes had been provided by all patients at the time of assessment for surgery.

Information on patients’ characteristics, primary tumor site, histology, and management of the disease (neoadjuvant or adjuvant chemotherapy and/or radiotherapy) was collected from the medical records.

According to the international guidelines (8, 9), follow-up of patients with sarcoma consisted of clinical and radiological examinations performed every 4 months in the first 2 years since the end of the last active treatment (surgery or radiotherapy or chemotherapy), then every 6 months for the following 3 years, and then annually until at least 10 years since the last treatment. Information on loss to follow-up was available through the consultation of certificates from the registry office. A patient was considered lost to follow-up if he/she failed to complete the follow-up program and had no information on the outcomes (recurrence and/or death). Every observation with an incomplete follow-up was censored at the date of the last contact (n = 36).

Three outcomes were analyzed: SSS, LR, and DR, defined as time from the date of diagnosis to the date of last follow-up for censored cases, to the date of death related to the soft tissue sarcoma and their treatments (complications, even rare), or to the date of first recurrence, respectively. In detail, death due to sarcoma was defined as any death where sarcoma was the first cause of death as obtained from the death certificates. Local relapse and distant metastasis were defined as radiologic or pathological manifestation of sarcoma recurrence within/contiguous to or distant from the initial tumor site, at least 2 months after the primary treatment.

### 2.2. Statistical analysis

#### 2.2.1. Model development

On the basis of prior medical knowledge, age at diagnosis, gender, histological subtype, FNCLCC (*Fédération Nationale des Centres de Lutte Contre le Cancer*) grading, tumor size (cm), tumor site (upper or lower limbs) and tumor depth (deep-seated or superficial STS), surgical margins (R0 or R1), and perioperative treatment (neoadjuvant/adjuvant radiotherapy and/or chemotherapy) were identified *a priori* as possible predictors.

The variable selection was performed using backward selection with a conservative *a priori* set criterion of p < 0.2 for the variable inclusion in the multivariable model. The backward selection was combined with a functional form selection for continuous variables using the multivariable regression splines algorithm (27, 28). Ignoring an effective treatment in prognostic model development would result in incorrect predictions of the outcome when applied to new untreated individuals (24), and treatment was thus always included in the final model. Clinically meaningful interactions were included in the model to identify those interactions that could improve the model fit. Regression coefficients from the final multivariable Cox proportional hazards (PH) models were used for the construction of nomograms predicting 5- and 10-year SSS and 2-, 5-, and 10-year LR and DR probability. Treatment was not plotted as the nomogram was intended to be used for the prediction of the probability of SSS, LR, and DR in untreated patients.

PH assumption was tested individually for each predictor and globally using a formal significance test based on the unscaled and scaled Schoenfeld residuals. In addition, graphical assessment of PH assumption was performed by plotting scaled Schoenfeld residual over time.

#### 2.2.2. Model validation

The models (including the variable selection) were internally validated using the bootstrap resampling technique with 1,000 resamples. The predictive performance was evaluated through the optimism-corrected discrimination and calibration at 5 and 10 years of follow-up for SSS and at 2, 5, and 10 years for LR and DR. The model discrimination was evaluated using the Harrell’s C-index (29, 30). The calibration was assessed by comparing the event probabilities predicted by the two models with the observed event probabilities. However, instead of the less precise Kaplan–Meier estimates for the observed event probabilities, we used hazard regression approach to estimate the actual survival probability as a function of the transformed predicted survival probability (31).

All analyses were performed using STATA version (Release 17.0, College Station, Texas) and R Statistical Software (Foundation for Statistical Computing, Vienna, Austria).

## 3. Results

In total, 517 patients with STS were included in the study. Characteristics of patients with STS included in the study are described in **Table 1**. Male-to-female ratio was 1.2:1, and the mean age at diagnosis was 54.83 years. All patients underwent surgery and, according to their clinico-pathological risk factors, neoadjuvant and/or adjuvant radiotherapy [n = 176 (34.0%)], chemotherapy [n = 36 (6.9%)], or both [n = 154 (29.8%)]. Of the 517 patients with STS, 306 (59.2%) had a local or distant metastasis and 221 (42.7%) had died from their disease. The median follow-up time was 5.6 years (interquartile range, 2.7–9.3) for SSS, 4.3 (interquartile range, 1.6–7.9) for LR, and 3.9 (interquartile range, 1.0–7.9) for DR. The SSS was 68.1% [95% confidence interval (CI), 63.8–72.1] at 5 years and 55.6% (95% CI, 50.5–60.3) at 10 years. LR-free survival was 79.1% (95% CI, 75.3–82.4) at 2 years, 71.1% (95% CI, 66.7–75.1) at 5 years, and 66.0% (95% CI, 60.7–70.7) at 10 years, whereas DR was 65.9% (95% CI, 61.6–69.9) at 2 years, 57.5% (95% CI, 53.0–61.8) at 5 years, and 52.1% (95% CI, 47.1–56.8) at 10 years.

**Table 1:** Baseline characteristics of the patients who were included in the model building (n = 517).

	N	%
**Age at diagnosis (mean, SD)**	54.83	16.90
**Age at surgery (mean, SD)**	54.94	16.88
**Gender**		
Male	284	54.93
Female	233	45.07
**Anatomical site**		
Lower limb	398	76.98
Upper limb	119	23.02
**Tumor depth**		
Deep-seated	499	96.52
Superficial	18	3.48
**FNCLCC grading**		
G1	41	7.93
G2	108	20.89
G3	368	71.18
**Tumor dimension in cm (median, IQR)**	7.50	5.00, 10.00
**Microscopic margins involvement** ^1^		
No	495	95.74
Yes	22	4.26
**Histological subtype**		
Leiomyosarcoma	87	16.83
Dedifferentiated liposarcoma	46	8.90
Myxoid liposarcoma	70	13.54
Malignant peripheral nerve sheath tumor	27	5.22
Myxofibrosarcoma	55	10.64
Synovial sarcoma	55	10.64
Undifferentiated pleomorphic sarcoma	117	22.63
Vascular sarcomas	36	6.96
Other	24	4.64
**Perioperative treatment**		
None	151	29.21
Chemotherapy	36	6.96
Radiotherapy	176	34.04
Combined chemotherapy and radiotherapy	154	29.79
**Chemotherapy setting**		
Neoadjuvant	102	53.68
Adjuvant	71	37.37
Neoadjuvant + adjuvant^2^	17	8.95
**Chemotherapy regimens**		
Anthracycline + ifosfamide	171	90.00
Other^3^ **Radiotherapy setting **Neoadjuvant Adjuvant	19123207	10.0037.2762.73
**Recurrence (local and/or distant)**		
No	211	40.81
Yes	306	59.19
Local recurrence	82	26.80
Distant metastases	153	50.00
Both	71	23.20
**Follow-up time for local recurrence (median, IQR)**	4.29	1.56, 7.92
**Follow-up time for distant recurrence (median, IQR)**	3.88	1.05, 7.90
**Survival status**		
Alive	273	52.80
Sarcoma-specific death	221	42.75
Death from other causes	23	4.45
**Follow-up time for SSS (median, IQR)**	5.60	2.74, 9.26

SD, standard deviation; IQR, interquartile range; FNCLCC, Fédération Nationale des Centres de Lutte Contre le Cancer; SSS, sarcoma-specific survival.

^1^ Margins involvement corresponded to R1 surgery.

^2^ Three preoperative cycles of chemotherapy followed by further two adjuvant cycles.

^3^ Other chemotherapy regimens comprise dacarbazine-based regimens, high-dose ifosfamide, and gemcitabine + docetaxel.

### 3.1. Sarcoma-specific survival

Results of univariable and multivariable Cox analysis for SSS are shown in **Table 2**. The final model for SSS included age at diagnosis, gender, surgical margins, FNCLCC grading, histology, therapy, and tumor size transformed using restricted cubic splines with three knots (at 2, 6, and 10). On the basis of the multivariable model (**Table 2**), the SSS decreased with increasing age, tumor size, FNCLCC grade, as well as for malignant peripheral nerve sheath tumor (MPNST) and undifferentiated pleomorphic sarcoma (UPS) and positive margins. On the other hand, female sex and all the remaining histological subtypes (e.g., dedifferentiated and myxoid sarcoma) were predictors of better SSS.

**Table 2:** Hazard ratios and 95% confidence intervals from the univariable and multivariable Cox proportional hazards models predicting 10-year sarcoma-specific survival.

	Univariable models		Multivariable model^4^	
	HR	95% CI	HR	95% CI
**Age**	1.01	1.01, 1.02	1.01	1.00, 1.02
**Gender**				
Male	1.00	–	1.00	–
Female	0.62	0.46, 0.82	0.64	0.47, 0.87
**Anatomical site**				
Lower limb	1.00	–	–	–
Upper limb	0.99	0.71, 1.38	–	–
**Tumor depth**				
Deep-seated	1.00	–	–	–
Superficial	0.61	0.23, 1.64	–	–
**FNCLCC grading**				
G1	1.00	–	1.00	–
G2	1.81	0.69, 4.77	1.97	0.79, 4.89
G3	4.94	2.03, 12.02	3.18	1.29, 7.84
**Tumor dimension**	1.03	1.02, 1.05		
Spline term 1^1^	–	–	1.93	1.53, 2.44
Spline term 2^2^	–	–	0.65	0.55, 0.76
**Microscopic margins involvement^3^**				
No	1.00	–	1.00	–
Yes	1.82	1.04, 3.20	1.94	0.95, 3.96
**Histological subtype**				
Leiomyosarcoma	1.00	–	1.00	–
Dedifferentiated liposarcoma	0.71	0.39, 1.28	0.47	0.24, 0.91
Myxoid liposarcoma	0.26	0.13, 0.52	0.30	0.15, 0.61
Malignant peripheral nerve sheath tumor	2.35	1.38, 3.98	2.19	1.35, 3.57
Myxofibrosarcoma	0.69	0.39, 1.24	0.60	0.32, 1.12
Synovial sarcoma	0.67	0.39, 1.15	0.80	0.47, 1.36
Undifferentiated pleomorphic sarcoma	1.38	0.92, 2.05	1.10	0.72, 1.67
Vascular sarcomas	0.53	0.26, 1.10	0.61	0.27, 1.40
Other	0.42	0.17, 1.06	0.38	0.14, 1.02
**Perioperative treatment**				
None	1.00	–	1.00	–
Chemotherapy	2.81	1.71, 4.61	1.38	0.83, 2.30
Radiotherapy	1.08	0.75, 1.57	0.67	0.45, 0.98
Combined chemotherapy and radiotherapy	1.26	0.86, 1.83	0.59	0.39, 0.90

HR, hazard ratio; CI, confidence interval; FNCLCC, Fédération Nationale des Centres de Lutte Contre le Cancer; margins involvement corresponded to R1 surgery.

^1^ Spline term 1: Untransformed tumor dimension in cm (dim1 ).

^2^ Spline term 2: $dim2=\frac{max{\left(0,\;\left(dim1-2\right)\right)}^{3}-{\left(10-6\right)}^{-1}\left\{max{\left(0,\;\left(dim1-6\right)\right)}^{3}\left(10-2\right)-max{\left(0,\;\left(dim1-10\right)\right)}^{3}\left(6-2\right)\right\}}{{\left(10-2\right)}^{2}}$ .

^3^ Margins involvement corresponded to R1 surgery.

^4^ To calculate the 5- and 10-year survival probability, use the following formula S_0_(t)^exp(xβ)^, where S_0_(t) is the baseline survival [ S_o_(5) = 0.9954442607398712 and S_o_(10) = 0. 992331263901129] and xβ is a weighted sum of the variables in the model, where the weights are the regression coefficients [i.e., log(HR)].

**Figure 1:**
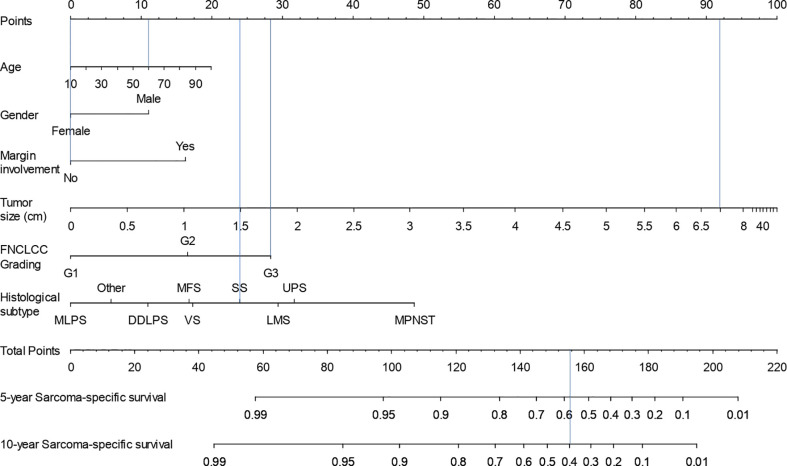
Nomogram for calculation of 5- and 10-year sarcoma-specific survival probability.

The 5- and 10-year SSS nomogram corresponding to the multivariate Cox regression model is presented in **Figure 1**. For example, a 60-year-old (11.5 points) female patient (0 points) with a 7-cm (92 points) FNCLCC grade 3 (28.5 points) synovial sarcoma (23.5 points) and no surgical margins involvement (0 points) would have a 5-year SSS probability of 0.58 and a 10-year SSS probability of 0.40.

**Figure 2:**
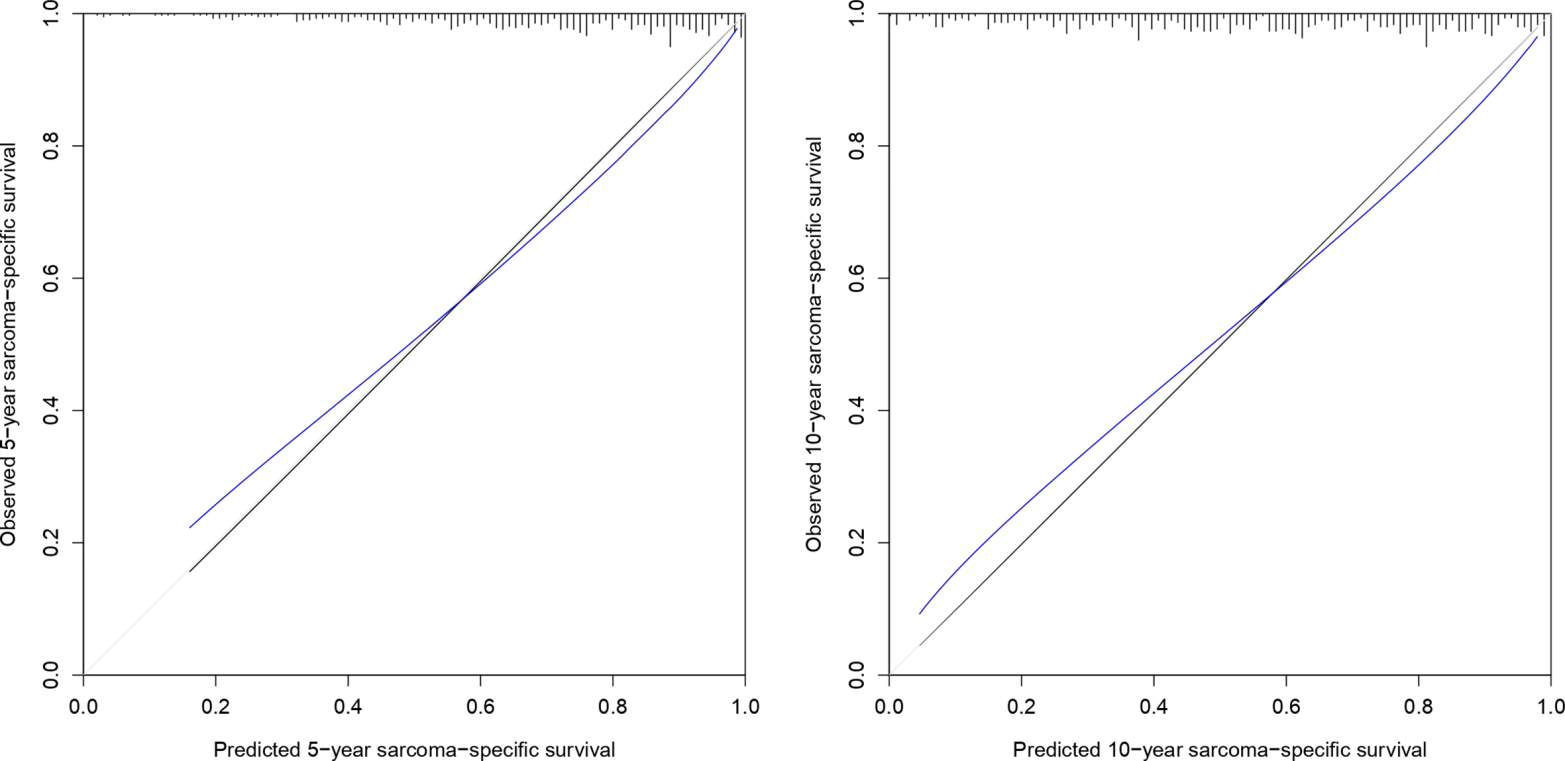
Calibration plot of 5- and 10-year sarcoma-specific survival probability based on 1,000 bootstrapped resamples. Black lines indicate observed calibration, gray lines indicate ideal calibration, and blue lines indicate optimism-corrected calibration.

The optimism-corrected C-index for the 5- and 10-year SSS was 0.73 and 0.72, respectively. The calibration plots for SSS at 5 and 10 years of follow-up are presented in **Figure 2**. The apparent predicted probabilities were close to the observed probabilities, indicating good calibration at both time points. However, small differences between the apparent and bias-corrected lines could indicate overfitting, which was more pronounced for the lower predicted probabilities (i.e., <0.4).

The global test indicated violations of the PH assumption for age, vascular sarcoma histology subgroup, and FNCLCC grade 3 (**Supplementary Materials**: **Table S1**). However, a close inspection of the smoothed residual plots for individual predictors not meeting the PH assumption showed only minimal departures from the straight line (see **Supplementary Materials**: **Figure S1**).

### 3.2. Local recurrence

**Figure 3:**
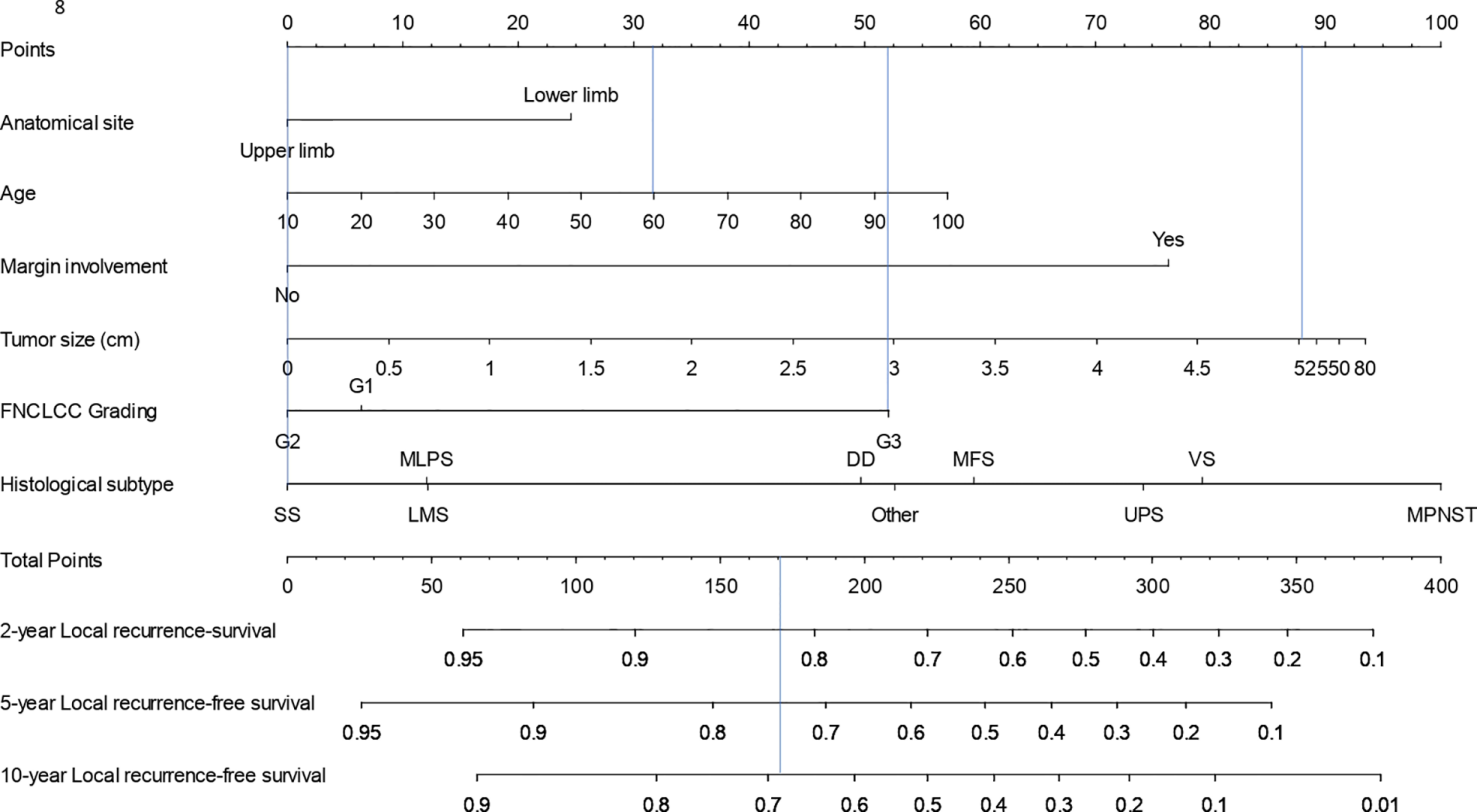
Nomogram for calculation of 2-, 5-, and 10-year local recurrence-free survival probability.

Results of univariable and multivariable Cox model for LR are shown in **Table 3**. The final model for LR included age at diagnosis, anatomical site, surgical margins, FNCLCC grading, histology, therapy, and tumor size transformed using linear splines with one knot (at 5). The 2-, 5-, and 10-year LR nomogram is presented in **Figure 3**. The predicted LR-free survival probability for the same patient from the SSS example whose STS was located in the upper limb was 0.82, 0.74, and 0.68 and at 2, 5, and 10 years.

**Table 3:** Hazard ratios and 95% confidence intervals from the univariable and multivariable Cox proportional hazards models predicting 10-year local recurrence.

	Univariable models		Multivariable model^4^	
	HR	95% CI	HR	95% CI
**Age**	1.02	1.00, 1.03	1.01	1.00, 1.02
**Gender**				
Male	1.00	–	–	–
Female	0.89	0.64, 1.23		
**Anatomical site**				
Lower limb	1.00	–	1.00	–
Upper limb	0.85	0.57, 1.26	0.74	0.49, 1.14
**Tumor depth**				
Deep-seated	1.00	–	–	–
Superficial	1.00	0.41, 2.43	–	–
**FNCLCC grading**				
G1	1.00	–	1.00	–
G2	0.85	0.40, 1.78	0.93	0.43, 1.98
G3	1.49	0.78, 2.84	1.73	0.85, 3.52
**Tumor dimension**	1.01	0.99, 1.03	–	–
Spline term 1^1^	–	–	1.23	0.96, 1.60
Spline term 2^2^	–	–	0.81	0.62, 1.06
**Microscopic margins involvement^3^**				
No	1.00	–	1.00	–
Yes	2.31	1.25, 4.28	2.50	1.31, 4.78
**Histological subtype**				
Leiomyosarcoma	1.00	–	1.00	–
Dedifferentiated liposarcoma	2.15	1.11, 4.18	1.57	0.78, 3.16
Myxoid liposarcoma	0.91	0.45, 1.84	1.00	0.47, 2.10
Malignant peripheral nerve sheath tumor	2.78	1.30, 5.94	2.88	1.32, 6.27
Myxofibrosarcoma	1.79	0.92, 3.52	1.77	0.89, 3.51
Synovial sarcoma	0.75	0.33, 1.68	0.86	0.38, 1.98
Undifferentiated pleomorphic sarcoma	2.22	1.26, 3.91	2.11	1.18, 3.77
Vascular sarcomas	2.09	1.03, 4.23	2.24	1.09, 4.62
Other	1.35	0.53, 3.43	1.63	0.64, 4.16
**Perioperative treatment**				
None	1.00	–	1.00	–
Chemotherapy	1.00	0.51, 1.97	0.67	0.33, 1.38
Radiotherapy	0.88	0.60, 1.29	0.66	0.44, 0.97
Combined chemotherapy and radiotherapy	0.50	0.31, 0.78	0.36	0.22, 0.59

HR, hazard ratio; CI, confidence interval; FNCLCC, Fédération Nationale des Centres de Lutte Contre le Cancer.

^1^ Spline term 1: Untransformed tumor dimension in cm ( dim1 ).

^2^ Spline term 2: dim2=(dim1>5)(dim1−5).

^3^ Margins involvement corresponded to R1 surgery.

^4^ To calculate the 5- and 10-year survival probability, use the following formula: S_0_(t)^exp(xβ)^ , where S_0_(t) is the baseline survival [ S_o_(2) = 0.9622265585818235, S_o_(5) = 0.9428302707056571, and S_o_(10) = 0.9280055692224253] and xβ is a weighted sum of the variables in the model, where the weights are the regression coefficients [i.e., log(HR)].

**Figure 4:**
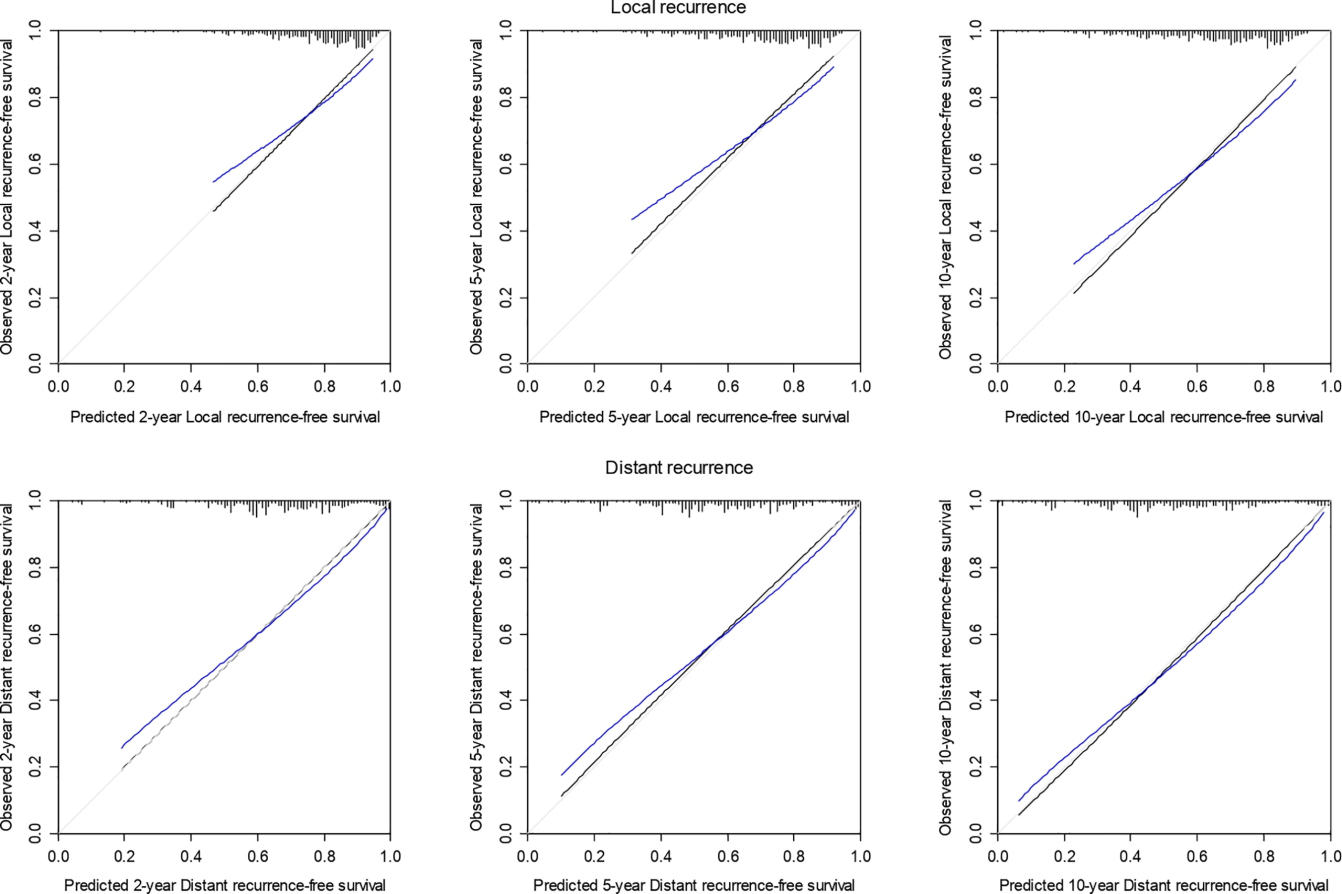
Calibration plot of 2-, 5-, and 10-year local and distant recurrence-free survival probability based on 1,000 bootstrapped resamples. Black lines indicate observed calibration, gray lines indicate ideal calibration, and blue lines indicate optimism-corrected calibration.

The optimism-corrected C-index for LR at 2, 5, and 10-years was 0.65, 0.64, and 0.64, respectively. The calibration plot for LR at 2, 5, and 10 years of follow-up is presented in **Figure 4**. The apparent predicted probabilities were similar to observed probabilities, indicating good calibration. However, at all times, the bias-corrected curves were different from the observed ones, indicating overfitting.

The individual and global tests indicated no violations of the PH assumption (see **Supplementary Materials**: **Table S2**).

### 3.3. Distant recurrence

**Figure 5:**
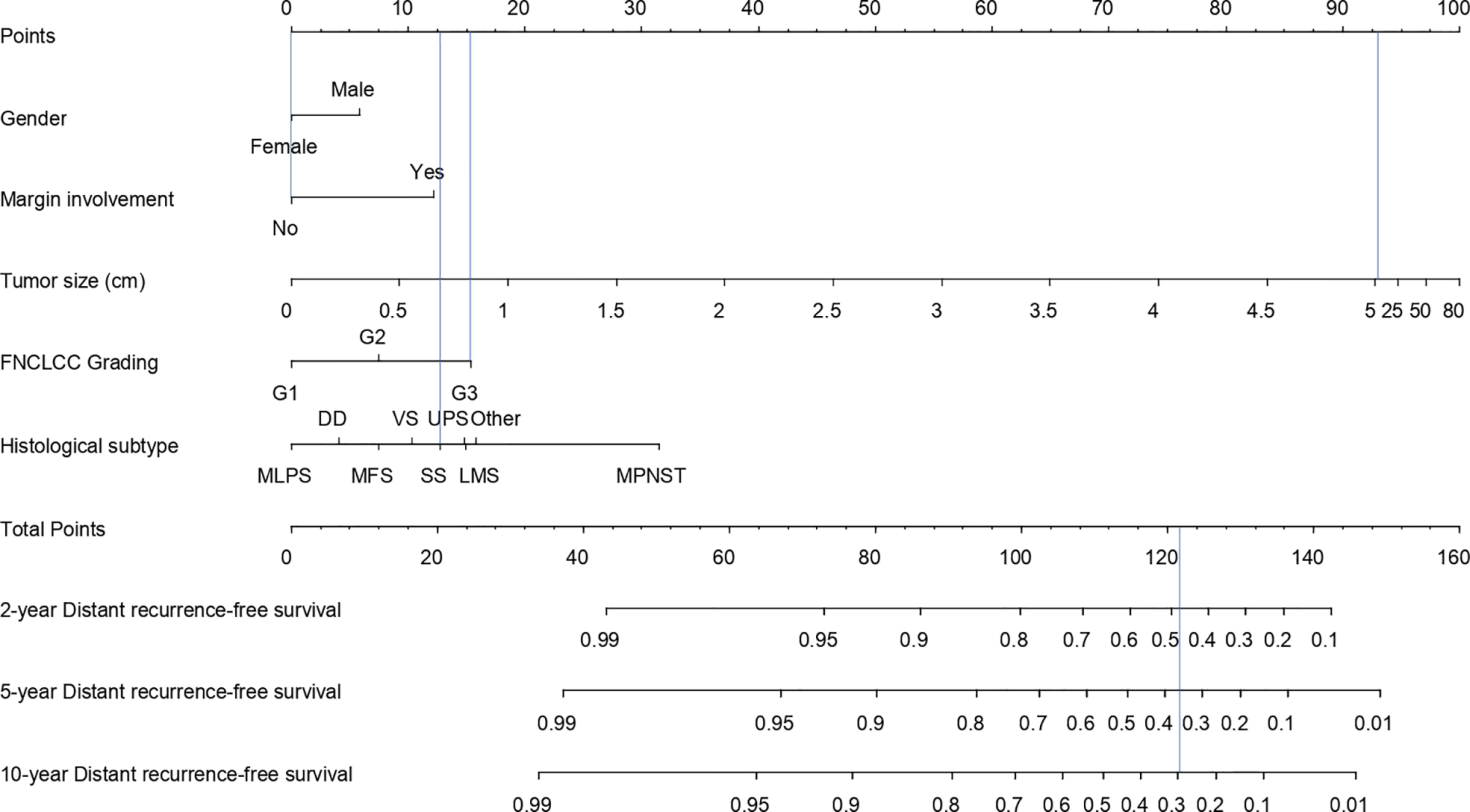
Nomogram for calculation of 5- and 10-year distant recurrence-free survival probability.

The univariable and multivariable Cox models predicting DR are presented in **Table 4**. The final model included gender, surgical margins, FNCLCC grading, histology, therapy, and tumor size transformed using linear splines with one knot (at 5). The 2-, 5-, and 10-year DR nomogram is presented in **Figure 5**, where we can see that the DR-free survival probability for the patient from the SSS example would be 0.48, 0.37, and 0.30 at 2, 5, and 10 years, respectively.

**Table 4:** Hazard ratios and 95% confidence intervals from the univariable and multivariable Cox proportional hazards models predicting 10-year distant recurrence.

	Univariable models		Multivariable model^4^	
	HR	95% CI	HR	95% CI
**Age**	1.00	0.99, 1.01	–	–
**Gender**				
Male	1.00	–	–	–
Female	0.68	0.52, 0.89	0.73	0.55, 0.96
**Anatomical site**				
Lower limb	1.00	–	–	–
Upper limb	1.06	0.78, 1.44	–	–
**Tumor depth**				
Deep-seated	1.00	–	–	–
Superficial	0.45	0.17, 1.20	–	–
**FNCLCC grading**				
G1	1.00	–	1.00	–
G2	1.44	0.69, 3.01	1.51	0.71, 3.18
G3	2.89	1.48, 5.64	2.32	1.14, 4.73
**Tumor dimension**	1.02	1.01, 1.04	–	–
Spline term 1^1^			2.77	1.83, 4.18
Spline term 2^2^			0.36	0.24, 0.55
**Microscopic margins involvement^3^**				
No	1.00	–	1.00	–
Yes	1.78	1.04, 3.06	1.94	1.01, 3.41
**Histological subtype**				
Leiomyosarcoma	1.00	–	1.00	–
Dedifferentiated liposarcoma	0.79	0.45, 1.37	0.55	0.31, 0.98
Myxoid liposarcoma	0.43	0.25, 0.75	0.44	0.25, 0.78
Malignant peripheral nerve sheath tumor	2.35	1.37, 4.01	2.47	1.42, 4.30
Myxofibrosarcoma	0.72	0.42, 1.25	0.66	0.39, 1.15
Synovial sarcoma	0.88	0.53, 1.45	0.88	0.53, 1.47
Undifferentiated pleomorphic sarcoma	1.20	0.81, 1.79	0.99	0.66, 1.49
Vascular sarcomas	0.75	0.41, 1.38	0.78	0.42, 1.44
Other	1.00	0.52, 1.95	1.05	0.54, 2.05
**Perioperative treatment**				
None	1.00	–	1.00	–
Chemotherapy	2.29	1.43, 3.66	0.96	0.57, 1.60
adiotherapy	0.92	0.65, 1.29	0.60	0.42, 0.85
Combined chemotherapy and radiotherapy	1.01	1.01, 1.43	0.48	0.33, 0.69

HR, hazard ratio; CI, confidence interval; FNCLCC, Fédération Nationale des Centres de Lutte Contre le Cancer.

^1^ Spline term 1: Untransformed tumor dimension in cm ( dim1 ).

^2^ Spline term 2: dim2=(dim1>5)(dim1−5) .

^3^ Margins involvement corresponded to R1 surgery.

^4^ To calculate the 5- and 10-year survival probability, use the following formula: S_0_(t)^exp(xβ)^ , where S_0_(t) is the baseline survival [ S_o_(2) = 0.9970782782008056, S_o_(5) = 0.9959510001044745, and S_o_(10) = 0.9951339734767251] and xβ is a weighted sum of the variables in the model, where the weights are the regression coefficients [i.e., log(HR)].

The optimism-corrected C-index for DR was 0.68 at all years. The calibration plot for DR at 2, 5, and 10 years of follow-up is presented in **Figure 4**. The apparent predicted probabilities were similar to observed probabilities. There was some indication of overfitting at 2 and 5 years.

The global test, but not the individual ones, indicated violation of the PH assumption (**Supplementary Materials**: **Table S3**). There were also no obvious departures from the straight line in the smoothed residual plots (**Supplementary Materials**: **Figure S2**).

## 4. Discussion

In the present study, we developed and internally validated three novel nomograms predicting SSS, LR, and DR for patients with STS of the extremities who were surgically treated at Humanitas Cancer Center (Rozzano, Milan, Italy). The final SSS nomogram incorporated six major patient and tumor characteristics (age, gender, margin involvement, tumor size, FNCLCC grading, and histological type); the LR nomogram included age, anatomical site, margin involvement, tumor size, FNCLCC grading, and histological type; whereas the DR nomogram included gender, margin involvement, tumor size, FNCLCC grading, and histological type. By also including perioperative treatment in our models, we allow clinicians to calculate the probability of SSS, LR, and DR if a patient was to remain untreated, thus facilitating a better-informed individualized decision-making (24).

In 2002, Kattan et al. developed the first nomogram predicting SSS for patients with STS (32). This model was subsequently externally validated (33) and followed by many studies developing STS-specific models predicting SSS (12, 14–16, 19, 22) (**Table 4**). Whereas the model by Kattan et al. included all tumor sites (32), other models were limited to a specific histological subtype, such as synovial sarcoma (12, 16), liposarcoma (19), leiomyosarcoma (14), and MPNST (15). The most similar study to ours in terms of population was the study by Sekimizu et al. (22). The differences in the populations used for model development make a direct comparison of these models difficult. However, majority of these models, including ours, contain overlapping predictors, and it is thus not surprising that they exhibit similar discriminative performance. Of note, the model by Kattan et al. has been updated and validated for predicting 10-year SSS among patients with STS of the extremities (34). This model is somewhat similar to ours, but it does not include gender nor margins involvement, and a continuous tumor size was reduced to three arbitrary categories; whereas, in our model, we allowed for continuous non-linear association between tumor size and SSS. We did not include tumor depth among predictors because, in our population, STSs were mainly deep-seated and tumor site was not a predictor of SSS in our model. Whereas superficial STS account for a 20% of all STS (35), by excluding some histological subtypes that could have a superficial location (e.g., atypical lipomatous tumor/well-differentiated liposarcoma and dermatofibrosarcoma protuberans), we observed a low number of superficial STS in our cohort. Unlike most of the abovementioned studies, we also included perioperative treatment in the model, thus allowing for the correct predictions of the SSS probability when the model is applied to new untreated individuals. In one of the aforementioned studies, chemotherapy was included in the nomogram as yet another predictor (15). Because this study was not designed according to the causal inference principles, estimated probabilities of outcome under different treatments could be mistakenly interpreted as treatment effects and mislead both patients and clinicians.

Although the evidence on the efficacy of preoperative chemotherapy for STS of the extremities is still conflicting (36, 37), recent studies have demonstrated improved prognosis for patients treated with standard neoadjuvant chemotherapy (anthracycline + ifosfamide) (38, 39). For the post-operative chemotherapy, the evidence on the improved relapse-free survival is more consistent (40–42). Furthermore, a systematic review and meta-analysis examined the effects of pre- and post-operative radiation therapy, thus confirming a reduced risk of local relapse when radiotherapy is performed (43). These findings indicate that both pre- and post-operative chemotherapy and radiotherapy could be associated with adverse outcomes and will likely change the natural course of the disease for treated patients. To avoid incorrect predictions of the outcome when our model is applied to new untreated individuals, we explicitly modeled treatment (24). Given that both neoadjuvant or adjuvant treatments are usually defined at a multidisciplinary meeting preceding surgery (9) and are administered shortly before or after the surgery and far from the outcome of interest, for simplicity, we combined neoadjuvant/adjuvant therapy in our prognostic model. Furthermore, as only a few patients were treated with non-standard chemotherapy regimens, we did not separately model different treatment groups, thus avoiding potential problems with quasi-complete or complete separation due to the small number of subjects in the smallest treatment categories. However, a finer adjustment for treatment, by including a more granular information on the treatment type and/or regimen, could potentially adjust better for the treatment, especially if there are large differences in the effect of different treatment regimens on the outcome.

Our study is the first study predicting the LR and DR at 2, 5, and 10 years. Several previously published models focused on either LR (22, 23) or distant metastases (11, 22), but not on both, thus covering only a part of the entire relapsed population. Furthermore, the populations used to develop models predicting LR or DR differed between the previously published studies. These differences preclude a direct comparison of the models, and it is not obvious if difference in model performance between these studies is due to the different predictors, different populations, different outcome definition, or all of these factors (**Table 5**). Although the association between metastatic disease and SSS is intuitive and clearly reported in literature [8, 9], there is a large amount of conflicting reports on the possible association between LR and decreased SSS. However, it has been demonstrated that LR is associated with systemic diffusion of the disease and that the majority of patients developing a local relapse will die from the disease (5, 23, 44, 45). Moreover, the management of LR is often very complex and may not be curative. On the other hand, the development of distant metastases in patients with sarcoma is associated with a mixed prognosis. Whereas some patients have a median overall survival of 24 months, a selected group of patients with oligometastatic disease (primarily lung) can have a significant improvement in overall survival (median overall survival, 35–78 months) if adequately treated (46–49). According to the international guidelines, standard follow-up after surgery for STS is performed every 3–6 months for 2–3 years, then every 6 months for next 2 years, and then annually. After 10 years, the likelihood of developing a recurrence is small, and follow-up should be individualized. Knowing each patient’s probability of developing LR and/or DR at different time points would allow clinicians to optimize individual follow-up.

**Table 5:** Clinical studies on STS nomograms.

						C-index			
Nomogram	Population	N	Outcome	Predictors	Validation	SSS	OS	Relapse
Kattan MW (2002)^32^	STS any site after local recurrence	2,163	SSS	Age, histology, size, depth, location, grade	Internal 60 bootstraps	12 years: 0.77		–
Canter RJ (2008)^12^	SS of any site, surgical patients not receiving anthracycline-ifosfamide chemotherapy	196	SSS 3 and 5 years	Site, size, depth, variant (mono-/bi-phasic)	Internal bootstrap	0.773		–
Callegaro D (2016)^11^	STS of the extremities (patients with macroscopically complete surgical resections)	Training: 1,452 Validation: French: 420 Canadian: 1,436 UK: 444	OS DM 5 and 10 years	OS: age, size, grade, histology DM: size, grade, histology	External		F: 0.698 C: 0.775 U: 0.762	F: 0.652 C: 0.744 U: 0.749
van Praag VM (2017)^23^	High-grade STS of the extremities	766	OS LR 3, 5, and 10 years	Age, size, depth, histology, surgical margin, RT	Internal, leave-one-out cross validation		0.677	0.696
Wang W (2019)^14^	Limb LMS (SEER)	Training: 604 Test: 604	SSS OS 3 and 5 years	Race, grade, surgery type, size, stage (including M+), age	Internal split-sample	0.727	0.709	–
Yan P (2019)^15^	MPNST, any site (SEER)	Training: 689 Validation: 42	SSS OS 3 and 5 years	SSS: stage, site, surgery, CHT OS: age, histology, site, surgery, CHT, stage	External	0.722	0.700	–
Sekimizu M (2019)^22^	STS of the trunk and extremities	2827	LRFS DMFS SSS	Age, histology, size, grade, depth, site, nodal metastases, margins, gender	Internal 50 bootstraps	2 years: 0.75		LR: 0.73 DM: 0.70
Zeng Z (2020)^16^	SS, any site, (SEER)	Training: 612 Test: 262	SSS	Age, gender, grade, extent (including M+), size, site	Internal split-sample	5 years: 0.73		–
Ye L (2020)^19^	Extremity liposarcoma (SEER)	Training: 1,522 Test: 648	SSS OS	Age, size, grade, surgery, distant metastases, sex	Internal split-sample	3 years: 0.878 5 years: 0.877 8 years: 0.889	0.862 0.839 0.825	–
De Sanctis R (present study)	STS of the extremities	517	SSS LRFS DRFS	SSS: age, gender, margins, size, grade, histology LRFS: age, anatomical site, margins, size, grade, histology DRFS: gender, margins, size, grade, histology	Internal 1,000 bootstraps	5 years: 0.73 10 years: 0.72		LRFS: 2 years: 0.65 5 years: 0.64 10 years: 0.64 DRFS: 2 years: 0.68 5 years: 0.68 10 years: 0.68

SS, synovial sarcoma; LMS, leiomyosarcoma; MPNST, malignant peripheral nerve sheath tumor; SSS, sarcoma-specific survival; OS, overall survival; DFS, disease-free survival; LR, local recurrence; DM, distant metastasis; DR, distant recurrence; LRFS, local recurrence-free survival; DMFS or DRFS, distant metastasis/recurrence-free survival; M+, metastatic disease; RT, radiotherapy; CHT, chemotherapy; SES, socioeconomic status.

A potential limitation of the present study is its internal validation, which could potentially lead to an optimistic model performance compared with external validation (test of the model performance in an external sample of patients independent of the one on which the model was originally developed). According to our calibration plots, this seems particularly true for LR prediction at all years. Thus, external validation is recommended before these nomograms are used in different populations. In addition, the homogeneity of a population affected by a rare disease could represent a problem in the definition of the study population. Indeed, the distribution of predictors and outcomes could be slightly different when considering populations living around a high-volume hospital or referred to it because of higher complexity of disease. Humanitas Cancer Center is a referral center for sarcomas, and a relatively high number of patients treated at our Center are extra-regional (n = 211, 39.9%). However, for a disease as rare as sarcomas, with only few referral centers in Italy, the inclusion of extra-regional patients should not be a source of bias. In our study, the distributions of predictors among extra-regional and regional patients with sarcoma patients quite similar (**Supplementary Materials**: **Table S4**), and we point out that their inclusion did not lead to the biased prediction estimates. On the contrary, inclusion of both regional and extra-regional patients should improve the generalizability of our study population. Furthermore, in this study, we chose to analyze the SSS rather than the overall survival. Although overall survival includes all causes of death and is the most reliable and available survival measure, SSS has a more meaningful outcome for clinical decision-making. Other causes of death could preclude the outcome of interest, and not accounting for these competing events could introduce bias in the estimated SSS. However, only a few patients died from other causes (especially among those with histological subtypes with good prognosis) in our sample, and we point out that bias introduced by not accounting for the competing events is minimal.

In conclusion, we developed well-performing nomograms predicting 5- and 10-year probability of SSS and 2-, 5-, and 10-year probability of LR and DR. These nomograms can be used to predict outcome(s) for new untreated individuals. Estimates from our nomograms, combined with the relative treatment effects from randomized trials, allow clinicians to estimate absolute benefit of additional treatment and could better inform their decision-making. However, external validation is necessary for these nomograms before being used in the clinical practice.

## Data availability statement

The dataset generated and analyzed during the current study will be available in the ZENODO repository as soon as the article will be published and available from the corresponding author upon reasonable request.

## Ethics statement

The study protocol involving human participants reviewed and approved by Humanitas Research Hospital Ethics Committee. The patients/participants provided their written informed consent to participate in this study.

## Author contributions

RD: conceptualization, methodology, investigation, writing - original draft, writing - review and editing, and visualization; RZ: methodology, writing - original draft, writing - review and editing; AS: investigation, writing - original draft, writing - review and editing, supervision. All authors contributed to the article and approved the submitted version.

## Funding

This work was supported by 5x1000 funding (National Taxation devolved to Research or Charity)

## Conflict of interest

RD: honoraria for advisory board consultancy from Novartis (outside the submitted work). AS: honoraria for advisory board consultancy from Bristol-Myers-Squibb, Servier, Gilead, Pfizer, EISAI, Bayer, Merck Sharp & Dohme, Arqule/Sanofi, Takeda, Roche, Abb-vie, Amgen, Celgene, Servier, Astrazeneca, Eli-Lilly, Sandoz, and Novartis (all outside the submitted work). RZ has no relevant affiliations or financial involvement with any organization or entity with a financial interest in or financial conflict with the subject matter or materials discussed in the manuscript. This includes employment, consultancies, honoraria, stock ownership or options, expert testimony, grants or patents received or pending, or royalties.

## Publisher’s note

All claims expressed in this article are solely those of the authors and do not necessarily represent those of their affiliated organizations, or those of the publisher, the editors and the reviewers. Any product that may be evaluated in this article, or claim that may be made by its manufacturer, is not guaranteed or endorsed by the publisher.

## Supplementary material

Supplementary Figure 1 Scaled Schoenfeld residuals plots over time for sarcoma-specific survival.

Supplementary Figure 2 Scaled Schoenfeld residuals plots over time for distant recurrence.

Supplementary Table 1 Test of proportional hazards assumption for sarcoma-specific survival.

Supplementary Table 2 Test of proportional hazards assumption for local recurrence.

Supplementary Table 3 Test of proportional-hazards assumption for distant recurrence.

Supplementary Table 4 Baseline characteristics of the patients from the Lombardy region compared with the extra-regional patients.
