# Supplementary material for: Nomograms predicting local and distant recurrence and disease-specific mortality for R0/R1 soft tissue sarcomas of the extremities
Source: Front Oncol. 2022 Sep 20;12:941896. doi: 10.3389/fonc.2022.941896 (PMC9530899; doi:10.3389/fonc.2022.941896)
Supplement: Supplementary Figure 1 — Scaled Schoenfeld residuals plots over time for sarcoma-specific survival. [file DataSheet_1.docx]

Table S1. Test of proportional-hazards assumption for SSS

|  | Time: Log(t) | | | | |
| --- | --- | --- | --- | --- | --- |
|  | rho | chi2 | df | | Prob>chi2 |
| Age | -0,13112 | 5,20 | 1 | | 0,0226 |
| Gender | 0,02829 | 0,21 | 1 | | 0,6464 |
| Microscopic margins involvement | -0,08729 | 2,47 | 1 | | 0,1162 |
| Tumor dimension | | | | | |
| Spline term 1 | -0,01912 | 0,09 | | 1 | 0,7696 |
| Spline term 2 | 0,02178 | 0,11 | | 1 | 0,7399 |
| Histological subtype |  |  | |  |  |
| Leiomyosarcoma | . | . | | 1 | . |
| Dedifferentiated liposarcoma | -0,08747 | 1,91 | | 1 | 0,1670 |
| Myxoid liposarcoma | -0,08113 | 1,43 | | 1 | 0,2323 |
| Malignant peripheral nerve sheath tumor | 0,03433 | 0,22 | | 1 | 0,6366 |
| Myxofibrosarcoma | -0,04429 | 0,46 | | 1 | 0,4959 |
| Synovial sarcoma | -0,00624 | 0,01 | | 1 | 0,9236 |
| Undifferentiated pleomorphic sarcoma | -0,11328 | 2,90 | | 1 | 0,0887 |
| Vascular sarcomas | -0,22965 | 14,86 | | 1 | 0,0001 |
| Other | -0,03011 | 0,22 | | 1 | 0,6427 |
| FNCLCC grading |  |  |  | |  |
| G1 | . | . | 1 | | . |
| G2 | -0,04878 | 0,53 | 1 | | 0,4675 |
| G3 | -0,14288 | 4,99 | 1 | | 0,0255 |
| Perioperative treatment |  |  |  | |  |
| None | . | . | 1 | | . |
| Chemotherapy | -0,00033 | 0,00 | 1 | | 0,9959 |
| Radiotherapy | 0,08175 | 1,89 | 1 | | 0,1697 |
| Combined chemotherapy and radiotherapy | 0,03364 | 0,28 | 1 | | 0,5991 |
| Global test |  | 41,88 | 18 | | 0,0011 |

****Figure S1. Scaled Schoenfeld residuals plots over time for SSS

Table S2. Test of proportional-hazards assumption for the local recurrence

|  | Time: Log(t) | | | | |
| --- | --- | --- | --- | --- | --- |
|  | rho | chi2 | df | | Prob>chi2 |
| Age | 0.01274 | 0.03 | 1 | | 0.8581 |
| Anatomical site | 0.02523 | 0.13 | 1 | | 0.7186 |
| Microscopic margins involvement | -0.05281 | 0.55 | 1 | | 0.4594 |
| Tumor dimension |  |  | |  |  |
| Spline term 1 | -0.04326 | 0.42 | | 1 | 0.5166 |
| Spline term 2 | 0.03792 | 0.32 | | 1 | 0.5716 |
| Histological subtype |  |  | |  |  |
| Leiomyosarcoma | - | - | | 1 | - |
| Dedifferentiated liposarcoma | 0.06553 | 0.96 | | 1 | 0.3280 |
| Myxoid liposarcoma | 0.03249 | 0.22 | | 1 | 0.6389 |
| Malignant peripheral nerve sheath tumor | -0.02152 | 0.08 | | 1 | 0.7714 |
| Myxofibrosarcoma | 0.02514 | 0.13 | | 1 | 0.7182 |
| Synovial sarcoma | 0.03634 | 0.30 | | 1 | 0.5839 |
| Undifferentiated pleomorphic sarcoma | -0.05775 | 0.66 | | 1 | 0.4153 |
| Vascular sarcomas | -0.00408 | 0.00 | | 1 | 0.9512 |
| Other | -0.01247 | 0.03 | | 1 | 0.8581 |
| FNCLCC grading |  |  |  | |  |
| G1 | - | - | 1 | | -- |
| G2 | -0.03328 | 0.18 | 1 | | 0.6716 |
| G3 | -0.10305 | 1.85 | 1 | | 0.1739 |
| Perioperative treatment |  |  |  | |  |
| None | - | - | 1 | | . |
| Chemotherapy | 0.10532 | 2.02 | 1 | | 0.1553 |
| Radiotherapy | 0.12346 | 2.53 | 1 | | 0.1119 |
| Combined chemotherapy and radiotherapy | 0.08337 | 1.33 | 1 | | 0.2481 |
| Global test |  | 12.94 | 18 | | 0.7954 |

Table S3. Test of proportional-hazards assumption for the distant recurrence

|  | Time: Log(t) | | | |  |
| --- | --- | --- | --- | --- | --- |
|  | rho | chi2 | df | Prob>chi2 | |
| Gender | -0.05878 | 0.96 | 1 | 0.3272 | |
| Microscopic margins involvement | -0.06928 | 1.41 | 1 | 0.2347 | |
| Tumor dimension |  |  |  |  | |
| Spline term 1 | 0.06049 | 1.20 | 1 | 0.2742 | |
| Spline term 2 | -0.05922 | 1.14 | 1 | 0.2852 | |
| Histological subtype |  |  |  |  | |
| Leiomyosarcoma | - | - | 1 | - | |
| Dedifferentiated liposarcoma | 0.01893 | 0.11 | 1 | 0.7430 | |
| Myxoid liposarcoma | 0.13110 | 3.73 | 1 | 0.0535 | |
| Malignant peripheral nerve sheath tumor | 0.05249 | 0.77 | 1 | 0.3805 | |
| Myxofibrosarcoma | 0.02417 | 0.14 | 1 | 0.7108 | |
| Synovial sarcoma | 0.06586 | 1.04 | 1 | 0.3087 | |
| Undifferentiated pleomorphic sarcoma | -0.07164 | 1.41 | 1 | 0.2347 | |
| Vascular sarcomas | 0.01561 | 0.06 | 1 | 0.8121 | |
| Other | -0.00434 | 0.00 | 1 | 0.9489 | |
| FNCLCC grading |  |  |  |  | |
| G1 | - | - | 1 | - | |
| G2 | 0.06631 | 1.22 | 1 | 0.2692 | |
| G3 | -0.04301 | 0.49 | 1 | 0.4845 | |
| Perioperative treatment |  |  |  |  | |
| None | - | - | 1 | - | |
| Chemotherapy | -0.03587 | 0.41 | 1 | 0.5205 | |
| Radiotherapy | 0.09576 | 2.46 | 1 | 0.1165 | |
| Combined chemotherapy and radiotherapy | 0.09321 | 2.22 | 1 | 0.1366 | |
| Global test |  | 31.67 | 17 | 0.0166 | |

Figure S2. Scaled Schoenfeld residuals plots over time for DR

Table S4. Baseline characteristics of the patients from the Lombardy region compared with the extra-regional patients

|  | Lombardy Region  N=306 | | Extra-regional patients  N=211 | |  |
| --- | --- | --- | --- | --- | --- |
|  | N | % | N | % | p |
| **Age at diagnosis (mean, SD)** | 57.03 | 16.90 | 51.64 | 16.41 | 0.0003 |
| **Age at surgery (mean, SD)** | 57.14 | 16.88 | 51.75 | 16.41 | 0.0003 |
| **Gender** |  |  |  |  |  |
| Male | 167 | 54.58 | 117 | 55.45 | 0.844 |
| Female | 139 | 45.42 | 94 | 44.55 |  |
| **Anatomical site** |  |  |  |  |  |
| Lower limb | 228 | 74.51 | 170 | 80.57 | 0.108 |
| Upper limb | 78 | 25.49 | 41 | 19.43 |  |
| **Tumor depth** |  |  |  |  |  |
| Deep-seated | 300 | 98.04 | 199 | 94.31 | 0.023 |
| Superficial | 6 | 1.96 | 12 | 5.69 |  |
| **FNCLCC grading** |  |  |  |  |  |
| G1 | 21 | 6.86 | 20 | 9.48 | 0.130 |
| G2 | 57 | 18.63 | 51 | 24.17 |  |
| G3 | 228 | 74.51 | 140 | 66.35 |  |
| **Tumor dimension in cm (median, IQR)** | 8 | 5.4, 12 | 7 | 5, 10 | 0.0119 |
| **Microscopic margins involvement**^1^ |  |  |  |  |  |
| No | 291 | 95.10 | 204 | 96.68 | 0.380 |
| Yes | 15 | 4.90 | 7 | 3.32 |  |
| **Histological subtype** |  |  |  |  |  |
| Leiomyosarcoma | 41 | 13.40 | 46 | 21.80 | 0.016 |
| Dedifferentiated liposarcoma | 36 | 11.76 | 10 | 4.74 |  |
| Myxoid liposarcoma | 37 | 12.09 | 33 | 15.64 |  |
| Malignant peripheral nerve sheath tumor | 15 | 4.90 | 12 | 5.69 |  |
| Myxofibrosarcoma | 35 | 11.44 | 20 | 9.48 |  |
| Synovial sarcoma | 38 | 12.42 | 17 | 8.06 |  |
| Undifferentiated pleomorphic sarcoma | 69 | 22.55 | 48 | 22.75 |  |
| Vascular sarcomas | 24 | 7.84 | 12 | 5.69 |  |
| Other | 11 | 3.59 | 13 | 6.16 |  |
| **Perioperative treatment** |  |  |  |  |  |
| None | 83 | 27.12 | 68 | 32.23 | 0.421 |
| Chemotherapy | 21 | 7.19 | 15 | 7.11 |  |
| Radiotherapy | 103 | 33.66 | 73 | 34.60 |  |
| Combined chemotherapy and radiotherapy | 99 | 32.03 | 55 | 26.07 |  |
| **Recurrence (local and/or distant)** |  |  |  |  |  |
| No | 126 | 41.18 | 85 | 40.28 | 0.839 |
| Yes | 180 | 58.82 | 126 | 59.72 |  |
| **Survival status** |  |  |  |  |  |
| Alive | 164 | 53.59 | 109 | 51.66 | 0.740 |
| Sarcoma-specific death | 130 | 42.58 | 91 | 43.13 |  |
| Death from other causes | 12 | 3.92 | 11 | 5.21 |  |
| SD, Standard deviation; IQR, interquartile range; FNCLCC, *Fédération Nationale des Centres de Lutte Contre le Cancer;*  ^1^ Margins involvement corresponded to R1 surgery | | | | | |
